# High number of CD45RO+ tumor infiltrating lymphocytes is an independent prognostic factor in non-metastasized (stage I-IIA) esophageal adenocarcinoma

**DOI:** 10.1186/1471-2407-10-608

**Published:** 2010-11-05

**Authors:** Sandra Rauser, Rupert Langer, Sebastian Tschernitz, Peter Gais, Uta Jütting, Marcus Feith, Heinz Höfler, Axel Walch

**Affiliations:** 1 Institute of Pathology, Helmholtz Zentrum München, German Research Center for Environmental Health, Ingolstaedter Landstr. 1, 85764 Neuherberg, Germany; 2 Institute of Pathology, Technische Universität München, Ismaninger Str. 22, 81675 Munich, Germany; 3 Institute of Biomathematics and Biometry, Helmholtz Zentrum München, German Research Center for Environmental Health, Ingolstaedter Landstr. 1, 85764 Neuherberg, Germany; 4 Department of Surgery, Klinikum rechts der Isar, Technische Universität München, Ismaninger Str. 22, 81675 Munich, Germany

## Abstract

**Background:**

The validation of novel prognostic indicators is of greatest interest for the management of esophageal adenocarcinoma (Barrett's cancer), particularly for non-metastasized (stage I-IIA) disease. The prognostic role of tumor infiltrating T-lymphocytes (TILs) in Barrett's cancer has not been reported so far. Here we evaluated the impact of TILs on survival, recurrence, and metastasis in Barrett's cancer, particularly in stage I-IIA patients.

**Methods:**

The levels of the adaptive immune markers CD3, CD8, and CD45RO were analyzed by immunohistochemistry and image analysis in tissue microarrays consisting of tumor tissues of 118 patients with primary resected Barrett's cancer. The findings were correlated with clinicopathological parameters including patient outcome.

**Results:**

In multivariate analysis, a low density of intratumoral CD45RO+ immune cells was an independent unfavorable factor for disease-free survival in stages I-IIA patients (*P *= 0.004, RR = 4.7, 95% CI = 1.6-13.5) as well in the entire cohort (*P *= 0.048, RR = 2.0, 95% CI = 1.0-4.0). High CD3+ and CD45RO+ levels were associated with prolonged disease-free survival and overall survival as well with low recurrence rates of disease (*P *= 0.005 and *P *= 0.018, respectively). In addition, low CD3+ levels were correlated with a higher frequency of lymph node metastasis (*P *= 0.025).

**Conclusions:**

This study demonstrates that the density of CD45RO+ TILs is an independent prognostic factor in non-metastasized (stage I-IIA) Barrett's cancer patients and indicates an important role for the adaptive immunologic microenvironment. The inclusion of CD45RO+ density may help to improve the management of stage I-IIA Barrett's cancer.

## Background

Barrett's cancer (esophageal adenocarcinoma), most frequently arising from intestinal metaplasia, has rapidly increased in incidence over the last decades, faster than any other gastrointestinal malignancy [1,2]. Despite advances in the therapy of locally advanced tumors with multimodal treatment strategies including neoadjuvant chemo- or radiochemotherapy [3-5], it still remains one of the most deadly tumors in solid oncology. Therefore, there is the need of prognostic factors for improved individualized risk stratifications, and alternative treatment options, like targeted or immunologic therapeutic approaches.

Growth regulation and progression of cancer are not only influenced by the biological behavior of the tumor but also by the host defense mechanisms. Host immunity consists of a complex network of humoral and cellular components that interact with tumor cells and tumor stroma. On the cellular level, inflammatory elements encompass dendritic cells, macrophages, granulocytes and lymphocytes. Activated, memory- and cytotoxic tumor infiltrating T-lymphocytes (TILs) are considered to be manifestations of a specific host immune reaction against cancer cells, related to the cytotoxic activity and the production of growth modulating cytokines of TIL [6,7]. Several studies have demonstrated the presence of TILs in various solid human cancers, most recently showing the association of an abundance of CD3+, CD8+ or CD45RO+ lymphocytic tumor infiltration with a survival benefit for patients with gastric [8] and colorectal [9-11] cancer, as well as for patients with endometrial [12], cervical [13], ovarian [14,15], urothelial [16] and hepatocellular [17] carcinoma and melanoma [18]. Furthermore, it was argued that the type, density, and location of immune cells in colorectal cancer have prognostic values that are superior to and independent of those of the UICC-TNM classification [10,19]. However, the effect of TILs in the clinical course of Barrett's cancer is largely unknown. The validation of novel prognostic indicators are therefore of greatest interest for the management of esophageal adenocarcinoma, particularly for non-metastasized (stage I-IIA) disease.

The aim of this study was to evaluate the impact of TILs on survival, recurrence, and metastasis in primary resected Barrett's cancer, particularly in non-metastasized (stage I-IIA) patients.

## Methods

### Patient material

In total, 118 patients with adenocarcinomas of the distal esophagus [(Barrett's cancer associated with histopathologically identified Barrett's esophagus (according to WHO 2000)] [20] were enrolled into this study. The patients had undergone primary surgical resection without chemo- or radiotherapy at the Department of Surgery, Klinikum rechts der Isar, Technische Universität München between 1991 and 2004. For all patients formalin-fixed paraffin-embedded tissue from surgical resection was available and individual patient data including outcome were acquired with approval from the ethics committee of the Technische Universität München. All tumor tissue specimens were procured from patients giving written informed consent.

Mean age of the patients was 63.6 years (range, 33-83). Female/male ratio was 9/109. 47 tumors were UICC stage I, 18 stage IIA, 16 stage IIB, 28 stage III, and 8 stage IV. Lymph node metastases were absent in 65 cases (pN0M0) and present in 51 cases (pN1M0/1). The medium follow-up of the patients was 33 months for overall (range: 0.8 to 164 months) and 31 months for disease-free survival (range: 1.6 to 164 months).

### Tissue microarrays

Using a tissue microarray instrument (Beecher Instruments, Sun Prairie, Wisconsin, USA), three representative areas of each tumor (1.0 mm diameter) were removed from paraffin-embedded tissue blocks which had been prepared at the time of resection. Serial sections were cut for the purpose of immunohistochemistry and transferred to adhesive slides using the "paraffin-tape-transfer-system" according to manufacturer's instructions (Instrumedics, Hackensack, NJ, USA).

### Immunohistochemistry

Immunohistochemical stainings for CD3, CD8, and CD45RO were carried out using an automated stainer (Ventana Discovery, Tuscon, AZ, USA) and DAB Map kit (Ventana). Monoclonal antibodies against CD3 (total T-cell marker; NeoMarkers, Fremont, CA, USA) were used in dilution at 1:100, against CD8 (cytotoxic T-cell marker; Dako, Hamburg, Germany) in dilution at 1:50 and against CD45RO (memory T-cell marker; Dako, Hamburg, Germany) in dilution at 1:1200.

### Image analysis

Slides were analyzed with the use of an image-analysis workstation (SAMBA microscopic image processor; Meylan, France), the hardware and software of which have been first described by Brugal *et al *[21]. This system is fitted with a standard orthoplan microscope (Leica, Bensheim, Germany) and a colour TV-camera (JVC (KY-F30), 3-CCD, Tokyo, Japan). T-cells were analyzed in at least 10 randomly selected high-power fields (magnification x400 with the Leica microscope). Formal scoring (labeling index) for each antibody was then performed in one section for each TMA block. A labeling index resulted from the percentage of nuclear immunopositive area in relation to the total nuclear area of infiltrative tumor cells within the tissue. For feature extraction we applied the software running on SAMBA system [22].

### Statistical Analysis

Receiver operating characteristic (ROC) analyses were done to determine cut-off levels for labeling indices of TILs (CD3, CD8, CD45RO positive lymphocytes) that correlate with clinical outcome (overall survival). The optimization of the cut-off determination was done by significance. All cases were therefore classified into low- and high-density groups for each marker, that is CD3+ low, CD8+ low, and CD45RO+ low (low-density groups) and CD3+ high, CD8+ high, and CD45RO+ high (high-density groups). Correlations between parameters were performed using the Pearson's correlation for continuous variables, and for discrete variables the Chi square test or Fisher's exact test was used. Disease-free survival and overall survival were calculated from the date of surgical resection to the date of first recurrence, death, or last follow-up and the rates were determined according to the Kaplan-Meier method. Comparisons of survival curves were made using the log-rank test and multivariate analyses were done using the stepwise Cox regression analysis. Hazard ratios (risk ratios) were calculated univariate and multivariate and listed together with the confidence intervals. *P *values < 0.05 were considered statistically significant in all analyses, whereas *P *values of 0.05 to 0.1 were considered as trend. All statistical analyses were done using the SAS (SAS Institute, Cary, NC, USA) software package.

## Results

### Tumor infiltrating lymphocytes (TILs)

Immunohistochemical stainings were available for CD3 in 99 cases, for CD8 in 107 cases, and for CD45RO in 110 cases. Labeling indices of CD3+ ranged from 0.3-18.8 (mean 5.65), of CD8+ from 0.01-6.2 (mean 0.9) and of CD45RO+ levels from 0-32.1 (mean 2.1). There was a significant correlation between the TIL levels of CD3+ and CD8+ TILs (*P *< 0.001; r = 0.54), CD3+ and CD45RO+ TILs (*P *< 0.001, r = 0.4) and CD8+ and CD45RO+ TILs (*P *< 0.001; r = 0.53). Cut-off levels with prognostic relevance were 0.9 for CD3+, 0.5 for CD8+ and 2.0 for CD45RO+.

### Correlation between clinicopathologic features and TIL density

The clinical and pathological characteristics of the patients grouped by TIL density are summarized in Table 1. The CD3+ low group showed a higher frequency of lymph node metastasis than the corresponding high group (*P *= 0.025). Moreover, high CD3+ and high CD45RO+ levels were associated with less recurrence of disease (*P *= 0.005 and *P *= 0.018, respectively). A low density of CD3+ showed a trend with higher stages of UICC classification (*P *= 0.086). Nevertheless, by building the two groups of the early stages I-IIA versus the late stages IIB-IV within the UICC classification, a high level of CD3+ immune cells were significantly associated with early stages (*P *= 0.025). Furthermore, low levels of CD45RO+ immune cells showed a trend for an association with a higher frequency of lymph node metastasis (*P *= 0.065), and grading (*P *= 0.091). Apart from that there was no correlation of levels of TILs with the pT category, distant metastasis, grading and UICC classification.

**Table 1:** Clinical and pathological characteristics of the patients grouped by TIL density

	CD3+ TIL n = 99			CD8+ TIL n = 107			CD45RO+ TIL n = 110		
Characteristics	low	high	*P*-value	low	high	*P*-value	low	high	*P*-value
									
***Total number of patients***	42 (42.4%)	57 (57.6%)		50 (46.7%)	57 (53.3%)		17 (15.5%)	93 (84.5%)	
									
***Gender***			0.453			1.0			0.607
**male**	38 (38.4%)	54 (54.5%)		46 (43.0%)	53 (49.5%)		15 (13.6%)	87 (79.1%)	
**female**	4 (4.0%)	3 (3.0%)		4 (3.7%)	4 (3.7%)		2 (1.8%)	6 (5.5%)	
									
***Tumour stage***			0.298			0.688			0.353
**pT1**	15 (15.3%)	26 (26.5%)		20 (18.9%)	25 (23.6%)		6 (5.5%)	42 (38.5%)	
**pT2**	7 (7.1%)	12 (12.2%)		9 (8.5%)	12 (11.3%)		2 (1.8%)	18 (16.5%)	
**pT3**	20 (20.4%)	18 (18.4%)		21 (19.8%)	19 (17.9%)		9 (8.3%)	32 (29.4%)	
									
***Lymph node metastasis***			0.025			0.440			0.065
**pN0**	17 (17.3%)	35 (35.7%)		26 (24.5%)	31 (29.2%)		6 (5.5%)	54 (49.5%)	
**pN1**	25 (25.5%)	21 (21.4%)		24 (22.6%)	25 (23.6%)		11 (10.1%)	38 (34.9%)	
									
***Distant metastasis***			0.212			0.428			0.108
**M0**	37 (37.8%)	53 (54.1%)		45 (42.5%)	52 (49.1%)		14 (12.8%)	87 (79.8%)	
**M1**	5 (5.1%)	3 (3.1%)		5 (4.7%)	4 (3.8%)		3 (2.8%)	5 (4.6%)	
									
***Residual tumour***			0.070			0.276			0.125
**R0**	33 (34.4%)	51 (53.1%)		41 (39.4%)	51 (49.0%)		13 (12.1%)	81 (75.7%)	
**R1**	8 (8.3%)	4 (4.2%)		7 (6.7%)	5 (4.8%)		4 (3.7%)	9 (8.4%)	
									
***Grading***			0.273			0.696			0.091
**G1**	0 (0.0%)	2 (2.1%)		2 (1.9%)	1 (1.0%)		0 (0.0%)	5 (4.6%)	
**G2**	17 (17.5%)	27 (27.8%)		20 (19.0%)	26 (24.8%)		4 (3.7%)	42 (38.9%)	
**G3**	25 (25.8%)	26 (26.8%)		27 (25.7%)	29 (27.6%)		13 (12.0%)	44 (40.7%)	
									
***UICC classification***			0.086			0.776			0.156
**I**	11 (11.2%)	24 (24.5%)		16 (15.1%)	23 (21.7%)		4 (3.7%)	38 (34.9%)	
**II**	11 (11.2%)	19 (19.4%)		16 (15.1%)	17 (16.0%)		4 (3.7%)	28 (25.7%)	
**III**	15 (15.3%)	10 (10.2%)		14 (13.2%)	12 (11.3%)		6 (5.5%)	21 (19.3%)	
**IV**	5 (5.1%)	3 (3.1%)		4 (3.8%)	4 (3.8%)		3 (2.8%)	5 (4.6%)	
									
***Recurrence of disease***			0.005			0.209			0.018
**No**	13 (13.3%)	33 (33.7%)		21 (19.8%)	30 (28.3%)		4 (3.7%)	50 (45.9%)	
**Yes**	29 (29.6%)	23 (23.5%)		28 (26.4%)	27 (25.5%)		13 (11.9%)	42 (38.5%)	

### CD3+ and CD45RO+ TILs as predictors of patient survival

**Figure 1:**
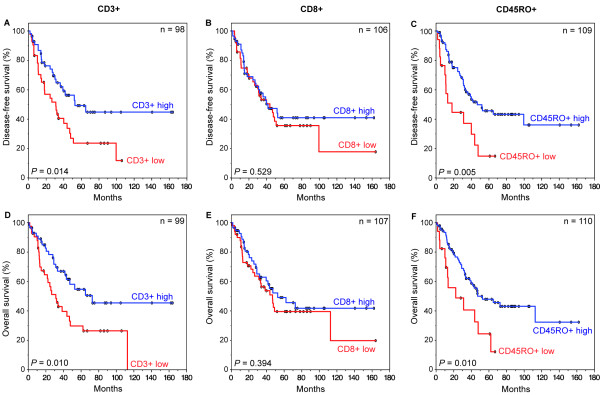
**Kaplan-Meier plots of disease-free (**A-C**) and overall (**D-F**) survival in primary resected Barrett's cancer patients according to CD3+ (**A **and **D**), CD8+ (**B **and **E**), and CD45RO+ (**C **and **F**) tumor infiltrating lymphocytes (TILs)**. In each cohort high and low TIL densities were plotted according to the corresponding cut-off values. Blue lines indicate patients with tumors containing high TIL densities, red lines indicate patients whose tumors had low densities of TILs. *P *values are calculated by log-rank tests. The 5-year disease-free survival (DFS) rate for patients with high CD3+ levels was 49.2% compared to 23.6% for patients with low levels of CD3+ TILs (overall survival (OS): 54.7% *versus *29.7%). Patients with high densities of CD45RO+ TILs had a 5-year DFS rate of 45.7% in contrast to 14.9% for patients with low densities of CD45RO+ (OS: 47.9% *versus *24.4%).

In Kaplan-Meier survival analysis, a correlation between higher infiltration levels and survival was observed for CD3+ and CD45RO+ lymphocytes, but not for CD8+ lymphocytes (Figure 1). There were significant differences in disease-free survival between patients with high CD3+ levels and low CD3+ levels (median 30.6 months *versus *21.4 months) (*P *= 0.014), as well between patients with high CD45RO+ densities and low CD45RO+ densities (median 31.7 months *versus *13.2 months, *P *= 0.005) (Figure 1A-C). Similar results were observed for overall survival: CD3+ high and CD45RO+ high groups had a more favorable outcome than the corresponding low groups (median CD3+ high: 33.0 months *versus *CD3+ low 25.1 months, median CD45RO+ high: 33.0 months *versus *13.6 months) (*P *= 0.010 each) (Figure 1D-F).

Univariate Cox proportional regression hazard models for disease-free survival showed a relative risk (RR) of 2.39 for patients whose tumors had a low density of CD45RO+ cells (95% confidence interval [CI] = 1.28-4.46, *P *= 0.007) (overall survival: RR = 2.28, 95% CI = 1.19-4.35, *P *= 0.013). Relative risks for disease-free and overall survival for patients with low densities of CD3+ were 1.96 (95% CI = 1.13-3.39, *P *= 0.016) and 2.06 (95% CI = 1.18-3.61, *P *= 0.012), respectively.

**Figure 2:**
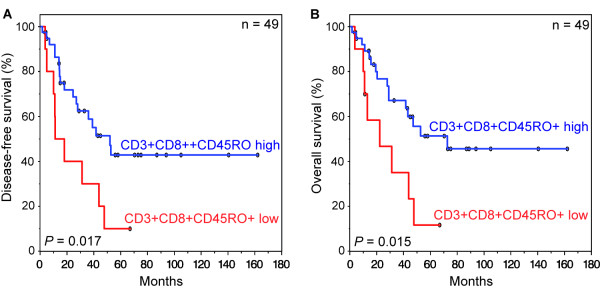
**Kaplan-Meier plots of disease-free (**A**) and overall (**B**) survival in primary resected Barrett's cancer patients as a function of tumor infiltrating lymphocyte (TIL) density**. Survival differences were found when high and low densities of CD3+, CD8+, and CD45RO+ were combined. In each cohort high and low TIL densities were plotted according to the corresponding cut-off values. Blue lines indicate patients with tumors containing high CD3+CD8+CD45RO+ densities, red lines indicate patients whose tumors had low densities of CD3+CD8+CD45RO+ TILs. *P *values are calculated by log-rank tests. The 5-year disease-free survival rate for patients with high levels of CD3+CD8+CD45RO+ TILs was 42.9% compared to 10.0% for patients with low levels of CD3+CD8+CD45RO+ TILs (overall survival: 51.3% *versus *11.7%).

We also found that the combined influence of high densities of CD3+CD8+CD45RO+ cells resulted in an improved disease-free (*P *= 0.017) and overall survival (*P *= 0.015) as compared to low levels of CD3+CD8+CD45RO+ (Figure 2). In the group of the patients with high levels of CD3+CD8+CD45RO+ TILs median disease-free survival time was 29.0 months as compared to 14.6 months for patients with low densities of combined CD3+CD8+CD45RO+ cells (overall survival: 33.0 *versus *17.7 months) (Figure 2). There was also a statistically significant prolonged survival, when high densities of CD3+CD45RO+ cells were combined (disease-free survival *P *= 0.004, overall survival *P *= 0.007). *P*-values for the combination of CD3+CD8+ cells were *P *= 0.055 for disease-free survival and *P *= 0.029 for overall survival. For the combination of CD8+CD45RO+ cells there was a trend in prolonged survival (disease-free and overall survival each *P *= 0.059).

Remarkably, in multivariate Cox regression analysis a high infiltration level of CD45RO+ lymphocytes was a significant independent prognostic factor beside pN- and M-category as well for disease-free survival (*P *= 0.048) as for overall survival (*P *= 0.028) (Table 2). Expression patterns of CD8+ and CD45RO+ TILs had no independent prognostic value, nor did the combination of CD3+CD8+CD45RO+ TILs.

**Table 2:** Multivariate analyses

	Disease-free survival			Overall survival		
**Variable**	***P*-value**	**RR**	**95% CI**	***P*-value**	**RR**	**95% CI**
						
***pN0 patients***						
**CD45RO+ (low)**	**0.004**	4.7	1.6-13.5	n. s.		
						
***All patients***						
**pN**	**< 0.0001**	4.7	2.5-9.0	**< 0.0001**	4.6	2.4-8.8
						
**M**	**0.011**	3.1	1.3-7.3	**0.023**	2.8	1.1-6.6
						
**CD45RO+ (low)**	**0.048**	2.0	1.0-4.0	**0.028**	1.1	1.0-1.2

Abbreviations: CI, confidence interval; n.s., not significant; RR, relative risk

### TIL as a predictor of regional lymph node metastasis

**Figure 3:**
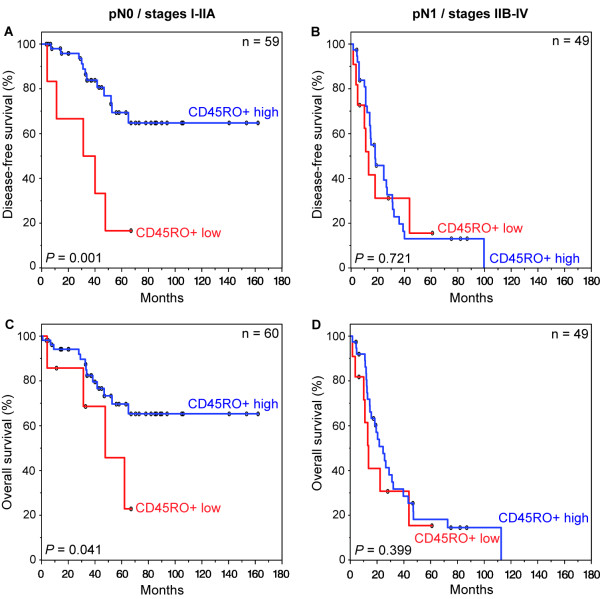
**Kaplan-Meier plots of disease-free (**A **and **B**) and overall (**C **and **D**) survival in primary resected Barrett's cancer patients according to CD45RO+ tumor infiltrating lymphocytes (TILs) and lymph node status**. (**A **and **C**) Survival rates in patients without regional lymph node metastases. (**B **and **D**) Survival rates in patients with presence of regional lymph node metastasis. In each cohort high and low TIL densities were plotted according to the corresponding cut-off values. Blue lines indicate patients with tumors containing high CD45RO+ levels, red lines indicate patients whose tumors had low densities of CD45RO+. *P *values are calculated by log-rank tests. The 5-year disease-free survival (DFS) rate in the group of the patients without lymph node metastases was 69.4% for high CD45RO+ levels compared to 16.7% for patients with low levels of CD45RO+ levels (overall survival (OS): 69.9% *versus *41.7%). In the group of the patients with lymph node metastases the 5-year DFS rate was 13.1% for patients with high densities of CD45RO+ in contrast to a rate of 15.6% for patients low densities of CD45RO+ (OS: 18.1% *versus *15.3%).

Disease-free and overall patient survival correlated with a high density of CD45RO+ TILs in pN0 patients, but not in pN1 patients (Figure 3). pN0 patients with a high density of CD45RO+ cells had a significant more favorable outcome than did patients with low CD45RO+ levels (disease-free survival: *P *= 0.001, overall survival: *P *= 0.041) (Figure 3A and 3C). In contrast, patients with lymph node metastasis (pN1) did not benefit from a high CD45RO+ level. There was no statistically significant difference between CD45RO+ levels and survival in pN1 patients (disease-free survival: *P *= 0.721, overall survival: *P *= 0.399) (Figure 3B and 3D).

Multivariate Cox regression analysis within the pN0-group revealed that low densities of CD45RO+ cells were independently associated with an unfavorable disease-free survival (*P *= 0.004, RR = 4.7, 95% CI = 1.6-13.5) (Table 2). In pN1 patients, distant metastasis was an independent prognostic factor (*P *= 0.002, RR = 3.6, 95% CI = 1.6-8.0).

Furthermore, the CD3+ low group showed a higher frequency of lymph node metastasis than the corresponding high group (*P *= 0.025) and for the CD45RO+ low group there was a trend for a higher presence of lymph node metastases (*P *= 0.065) (Table 1).

## Discussion

In this study, we analyzed the impact of tumor infiltrating lymphocytes (TILs) on survival, recurrence, and metastasis in primary resected esophageal adenocarcinoma (Barrett's cancer), particularly in stage I-IIA patients. The presence of high numbers of CD45RO+ lymphocytes was an independent predictor of increased survival particularly in non-metastasized (stage I-IIA) patients as well in the entire cohort. A high CD3+ level was further associated with prolonged survival and the absence of regional lymph node metastasis. Moreover, high infiltration levels of CD45RO+ and CD3+ T-cells were correlated with a lower rate of recurrence.

Similar observations were made in different cancer types. A study on gastric cancer demonstrated the independent prognostic value of the density of CD3+, CD8+, and CD45RO+ lymphocytes for regional lymph node metastasis and patient survival [8]. Studies on colorectal cancer showed that TILs predict survival better than conventional anatomical staging and that a high density of CD45RO+ lymphocytes is correlated with the absence of lymphatic vessel invasion [10,11]. In melanoma patients it was found that brisk TIL infiltration in tumor predicts sentinel lymph node metastasis [18] and in early-stage cervical cancer it was demonstrated that a high number of intraepithelial CD8+ lymphocytes is associated with the absence of lymph node metastasis [13]. Also, *in vivo *experiments and animal models have provided data supporting the role of TILs in cancer immunosurveillance [23,24]. Although the influence of lymphocytic infiltration has been studied in squamous cell cancer (SCC) of the esophagus [25-27], so far less information is available about the role of TILs in adenocarcinomas of the esophagus (Barrett's cancer).

To our knowledge, the current study is the first one to examine the prognostic influence of intratumoral CD3+ total T-lymphocytes, CD8+ cytotoxic T-lymphocytes, and CD45RO+ memory T-lymphocytes in a large cohort of primary resected Barrett's cancer patients (n = 118). In a previous study of a smaller series of human esophageal carcinomas (n = 70), which encompassed both squamous cell carcinomas and adenocarcinomas, the presence of a dense intratumoral CD8+ lymphocytic infiltration was associated with good prognosis independent from tumor stage and nodal stage [28]. However, we did not find CD8+ TILs to be a prognostic factor in Barrett's cancer. A recent work with a cohort of 106 adenocarcinomas of the esophagus failed to show a prognostic influence of CD3+ and CD8+ TILs [29], whereas in our cohort patients benefit from a high CD3+ lymphocytic infiltration. A possible explanation may be different methods applied to quantify TILs. In the study of Schumacher *et al *the number of CD8+ lymphocytes was counted by fluorescence microscopy and individual cases were classified in groups according to their location and quantification [28]. Zingg *et al *used high power field light microscopy and assessed the number of lymphocytes in two different locations, at the periphery and in the center of the tumors [29]. In our study, we used tissue microarrays with core biopsies taken randomized from the tumor area and the quantification of lymphocytic infiltration was done by image analysis. This approach has been demonstrated to be representative and has been used in different types of cancer [8-10, 12, 30].

Metastatic relapse attributable to the presence of tumor cells within lymph nodes is the most frequent cause leading to cancer-related death in patients with esophageal tumors [31]. In the current study, high levels of CD45RO+ TILs were significantly associated with improved survival in Barrett's cancer, on the one hand in the whole study cohort as well in the subgroup of non-metastasized patients. We also showed that high densities of CD45RO+ cells were an independent predictor of improved overall and disease-free survival. We found, that recurrence of disease less often occurred in the presence of high levels of CD45RO+ TILs. This observation is in line with the functional mechanism of memory T-lymphocytes, namely providing a more rapid and effective secondary immune response to previously encountered antigens [32]. Our data suggest that CD45RO+ T-lymphocytes within esophageal adenocarcinomas function not only locally but also systemically in tumor-draining lymph nodes to suppress micrometastasis.

Our results show that the presence of intratumoral CD45RO+ T-cells is associated with the lack of tumor metastases in the lymph nodes of Barrett's cancer patients. Because the absence of lymph node metastases is strongly associated with a better prognosis, patients with high CD45RO+ lymphocyte levels are likely to display an improved clinical outcome in Barrett's cancer. CD45RO+ and CD3+ TILs within Barrett's cancer may serve as criteria for selection and monitoring of patients for adjuvant immunotherapeutic strategies and can be a reliable prognostic marker to predict favorable outcome in patient subgroups. In our study, we focused on the T-lymphocytic cellular component, but additional studies are in need to elucidate the mechanisms and potency of host immunity. This is in particular concern for the purpose of the development of potential immunomodulatory or targeted cancer therapies that are highly required for the treatment of esophageal adenocarcinoma due to its aggressive behavior and the limited success of conventional therapy regimes. Our findings may provide a basis for risk adopted tumor treatment through predicting clinical outcome and definition of patient subgroups with different prognosis. The inclusion of CD45RO+ TIL density may help to improve the prognostication of stage I-IIA Barrett's cancer. Moreover, our results may be highly valuable in the context of future promising adjuvant immunotherapy and for potential immunomodulatory targeted therapy approaches.

## Competing interests

The authors declare that they have no competing interests.

## Authors' contributions

SR, RL and AW interpreted the data and drafted the manuscript. RL carried out assembly of tissue microarrays. ST and PG carried out the evaluation of the immunohistochemical stainings and image analysis and helped to draft the manuscript. UJ performed the statistical analyses and interpreted the data. MF participated in the design of the study and was responsible for collection and interpretation of clinical data. SR, RL, HH and AW conceived of the study and participated in its design and coordination. All authors red and approved the final manuscript.

## Pre-publication history

The pre-publication history for this paper can be accessed here:

http://www.biomedcentral.com/1471-2407/10/608/prepub
